# Evolutionary Novelty versus Exaptation: Oral Kinematics in Feeding versus Climbing in the Waterfall-Climbing Hawaiian Goby *Sicyopterus stimpsoni*

**DOI:** 10.1371/journal.pone.0053274

**Published:** 2013-01-04

**Authors:** Joshua A. Cullen, Takashi Maie, Heiko L. Schoenfuss, Richard W. Blob

**Affiliations:** 1 Department of Biological Sciences, Clemson University, Clemson, South Carolina, United States of America; 2 Aquatic Toxicology Laboratory, Saint Cloud State University, St. Cloud, Minnesota, United States of America; Laboratoire Arago, France

## Abstract

Species exposed to extreme environments often exhibit distinctive traits that help meet the demands of such habitats. Such traits could evolve independently, but under intense selective pressures of extreme environments some existing structures or behaviors might be coopted to meet specialized demands, evolving via the process of exaptation. We evaluated the potential for exaptation to have operated in the evolution of novel behaviors of the waterfall-climbing gobiid fish genus *Sicyopterus*. These fish use an “inching” behavior to climb waterfalls, in which an oral sucker is cyclically protruded and attached to the climbing surface. They also exhibit a distinctive feeding behavior, in which the premaxilla is cyclically protruded to scrape diatoms from the substrate. Given the similarity of these patterns, we hypothesized that one might have been coopted from the other. To evaluate this, we filmed climbing and feeding in *Sicyopterus stimpsoni* from Hawai’i, and measured oral kinematics for two comparisons. First, we compared feeding kinematics of *S. stimpsoni* with those for two suction feeding gobiids (*Awaous guamensis* and *Lentipes concolor*), assessing what novel jaw movements were required for algal grazing. Second, we quantified the similarity of oral kinematics between feeding and climbing in *S. stimpsoni*, evaluating the potential for either to represent an exaptation from the other. Premaxillary movements showed the greatest differences between scraping and suction feeding taxa. Between feeding and climbing, overall profiles of oral kinematics matched closely for most variables in *S. stimpsoni*, with only a few showing significant differences in maximum values. Although current data cannot resolve whether oral movements for climbing were coopted from feeding, or feeding movements coopted from climbing, similarities between feeding and climbing kinematics in *S. stimpsoni* are consistent with evidence of exaptation, with modifications, between these behaviors. Such comparisons can provide insight into the evolutionary mechanisms facilitating exploitation of extreme habitats.

## Introduction

Animals that live in, or travel through, extreme environments can be exposed to severe functional demands. However, species that have successfully penetrated such habitats often exhibit novel traits that help them to accommodate such demands [1], [2]. Gobioid fishes found in the streams of many volcanic, oceanic islands provide prominent examples of this pattern. Streams of volcanic islands are subject to a range of catastrophic disturbances including lava flows, hurricanes, and flash floods [3]. The ability of many gobioid species to persist in habitats subject to such extremes is facilitated by a complex, amphidromous life cycle [4]–[6]. Adult fish mate and deposit eggs in streams, but upon hatching the larvae are swept into the ocean where they develop for several months before returning to freshwater [4], [7], [8], providing an oceanic population reservoir from which disturbed streams can be repopulated [3].

To penetrate upstream habitats, many goby species must scale substantial waterfalls that can exceed tens of meters in height [9]. Such climbing is facilitated by the presence of a ventral sucker, common to all gobies, formed from the fusion of the pelvic fins [10], [11]. However, species of one goby genus, *Sicyopterus*, also exhibit a distinctive oral sucker that develops after larvae undergo a cranial metamorphosis that coincides with the return to freshwater, during which the mouth shifts from a terminal orientation to a subterminal position over the course of 36–48 h [12], [13]. The oral sucker facilitates use of a novel mechanism for accessing upstream habitats above waterfalls [9], [11], [14], [15]. This form of locomotion has been termed “inching” and requires alternate attachment of oral and pelvic discs to the rocky substrate, providing a slow, but steady, method of climbing that, in the Hawaiian species *S. stimpsoni,* allows individual fish to scale waterfalls up to 100 m tall [9], [10]. Juveniles from goby taxa that lack an oral disc, including Sicydiine outgroups to *Sicyopterus* such as the genera *Sicydium* and *Lentipes* [16], exhibit a different climbing behavior described as “powerburst” climbing. In this pattern, the pectoral fins are adducted before rapid undulation of the body, with no oral involvement in adhesion [9], [17]. Thus, it appears most parsimonious that the oral disc and cranial kinematics used by climbing *Sicyopterus* are derived, rather than basal features. Although the structural basis for the use of the mouth as a locomotor organ is clear in this genus, how did its novel locomotor strategy evolve?

In addition to its distinctive use of the mouth for locomotion, oral function during feeding also appears distinctive in *Sicyopterus* compared to other stream gobies, as exemplified by Hawaiian *S. stimpsoni*. A larval feeding strategy of capturing zooplankton changes to a juvenile strategy that involves scraping benthic diatoms from rocks [4], [18], [19]. In a broad sense, this behavior, like inching during waterfall climbing, also involves motion of the mouth against a substrate. With the mouth being used in generally similar ways for these different post-metamorphic behaviors, it is possible that, rather than evolving independently, jaw kinematics in one of these behaviors may simply have been coopted and implemented in a new behavior.

Numerous instances have been proposed in which a structure that had been used for one specific function appears to have been coopted for another function [20], suggesting this possibility in the behavioral evolution of *S. stimpsoni* and other members of the genus *Sicyopterus.* Such instances of evolutionary coopting have been termed “exaptations”[20]. In a classic example from the evolution of birds that was described in the paper that coined this term [20], feathers may have served originally to provide insulation and only later, after changes in feather shape and forelimb morphology, been coopted to serve a role contributing to sustained flight [20]. More recently proposed examples of exaptation have extended beyond structural features to include behavioral and biomechanical traits [21]–[24]. For example, juvenile chukar partridges display a behavior termed “wing-assisted incline running,” in which they flap small, immature wings in order to climb inclines. The discovery that such behaviors generate substantial lift suggests the potential that the functional capacities of even appendages with suboptimal wing morphology might have been coopted during the eventual evolution of flight [21]. In another example of the coopting of a motor behavior from one function to another, trap-jaw ants typically use the rapid closing strikes of their mandibles for prey capture, but can also use them to propel themselves into the air by simply reorienting strikes to be directed against the ground [22]. In a closer parallel to the gobiid fish system, previous studies have shown herbivorous benthic scraping abilities [25], [26] as well as station-holding abilities [27] among species of catfishes; however it is unclear how closely patterns of movement compare in such cases.

Although the feeding behavior of *S. stimpsoni* has been recognized as novel among Hawaiian gobiids, specific kinematic differences in comparison to other gobiid species have not been quantified. Moreover, while both climbing and feeding have been examined to some extent in *S. stimpsoni*, kinematic comparisons of these two behaviors that could help assess the potential for exaptation in this genus have not been performed. In this study, we measured the oral kinematics of climbing and feeding by *S. stimpsoni* for two sets of comparisons. First, to assess novel patterns of jaw motion required for algal grazing, we compared the feeding kinematics of *S. stimpsoni* with those previously published for two outgroup, suction feeding Hawaiian gobiids, *Awaous guamensis* and *Lentipes concolor* [28]. Second, in order to evaluate the potential for either feeding or climbing kinematics to represent the coopting of patterns of motion in the other behavior, we compared oral kinematics for these behaviors in *S. stimpsoni*. If the kinematics of these two behaviors were significantly different, it would be less likely that the performance of one function involved simple exaptation of the other. In contrast, if kinematics of these behaviors were similar, movements in one function may simply have been coopted for a different role in the other.

Highlighting the difficulty in formally identifying a trait as an exaptation, Lauder [29] identified four criteria for which evidence should be provided: (1) current utility of the trait, (2) selection for that trait in its current environment, (3) previous utility of the trait in an ancestral taxon for a different role than the current one, and (4) natural selection for that trait in the ancestral environment. Our previous studies have shown the utility of oral function in both climbing [9], [11], [30] and feeding [19] in *S. stimpsoni*, as well as selection on climbing performance [31], [32]. Because no species of *Sicyopterus* is known to use oral movements for one behavior (climbing or feeding) but not the other, it is difficult to establish a phylogenetic context that would point to one behavior being more likely ancestral, and it may not be possible to definitively evaluate which behavior might represent an exaptation. However, independent of which behavior came first, our primary goal is to consider whether the evolutionary mechanism of exaptation may have operated in this system, given the context of knowledge about utility and selection for feeding and climbing. The first step in such an assessment is to evaluate whether oral movements for climbing and feeding should be considered as the same trait, based on the extent of their similarity.

## Materials and Methods

### Ethics Statement

Permission for access to field sites and specimens was provided by the Division of Aquatic Resources, State of Hawai’i, coordinated by Dr. Robert Nishimoto. This study was carried out in strict accordance with the recommendations in the Guide for the Care and Use of Laboratory Animals of the National Institutes of Health. The protocol was approved by the Institutional Animal Care and Use Committee of Clemson University (Permit Number: 40061 and 2011-057).

### Specimen Acquisition and Filming of Feeding and Climbing

*Sicyopterus stimpsoni* (Gill 1860) were captured from Hakalau stream on the Island of Hawai’i by net while snorkeling. Fish ranged in size from 45 to 73 mm total length (mean ± s.e.m. 50.5±3.2 mm for feeding [*N* = 4 individuals], 64.0±7.2 for climbing [*N* = 3 individuals]), representing mid-sized, sexually mature individuals for this species. Within 2 hrs of capture, fish were transferred in stream water to lab facilities provided by the Hawai’i Division of Aquatic Resources in Hilo, Hawai’i, where they were housed in small groups (3–5 fish) in tanks of aerated stream water at ambient temperature (19°C). After overnight acclimation, filming proceeded over the following 2–3 days.

Feeding kinematics were filmed during the 2005 field season. To establish a grazing surface for feeding, glass microscope slides were submerged in shallow, sunny areas of streams. These slides were recovered after 1–3 days once a mild diatomaceous film had grown on the upper surface of the glass [19]. This provided an effectively transparent substrate through which oral kinematics could be filmed in ventral view. Each diatom-covered slide was placed in a 38 L glass aquarium with a clear bottom that was supported off the ground, allowing a mirror to be placed underneath at 45° to the tank bottom. *S. stimpsoni* were transferred individually to the aquarium and allowed to acclimate. Digitally synchronized lateral and ventral views of feeding were then filmed using two high-speed video cameras (500 Hz; Phantom V4.1, Vision Research, Wayne, NJ, USA).

Due to limits on the duration of field seasons and the availability of fish and equipment, synchronized lateral and ventral views of climbing could not be filmed until the 2011 field season. As a result, feeding and climbing kinematics were collected from different sets of fish. To allow both lateral and ventral views to be filmed, fish were stimulated to climb up a clear Plexiglas sheet [9]. Small groups of up to seven fish were placed in a holding tank containing stream water (60 cm wide, 45 cm long, 15 cm deep), from which the Plexiglas sheet emerged at an angle of 62°. This angle allowed stable attachment to the holding tank, and was very close to the 57° angle used in our previous study of climbing performance in adult *S. stimpsoni* [30]. To generate flow over the climbing surface, a siphon was used to direct a sheet of stream water down the Plexiglas from a 20 L bucket at 200 mL min^−1^. Fish were filmed at 200 Hz in lateral and ventral views once they had climbed 10 cm above the water level [33], using the same cameras as in feeding videos.

### Kinematic Analysis

**Figure 1:**
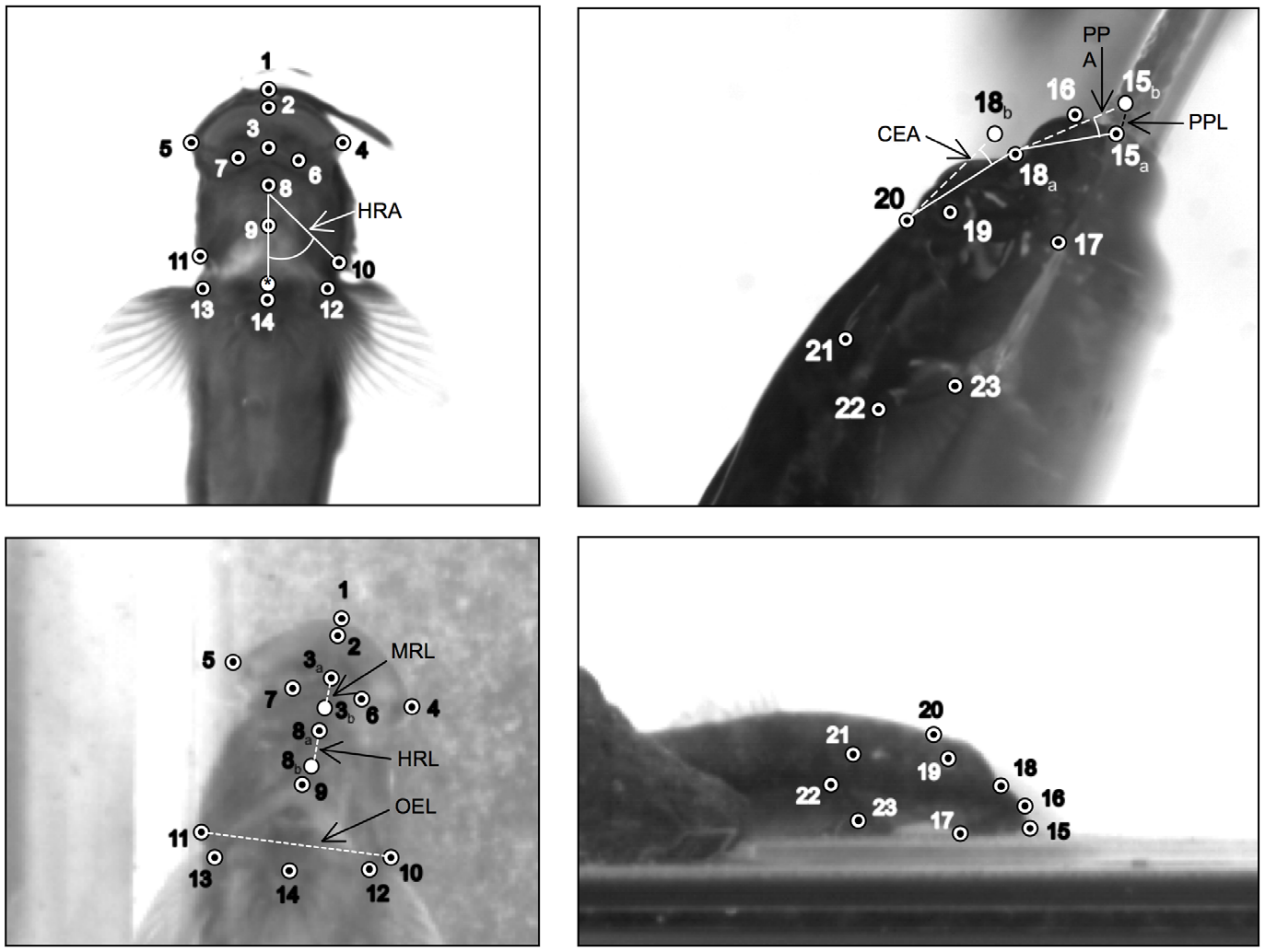
Still images of *S. stimpsoni* in (a) ventral and (b) lateral views, illustrating anatomical landmarks that were digitized to generate kinematic data. For ventral view (a), labeled points are as follows: (1) anterior edge of upper lip, (2) anterior tip of inner edge of upper lip, (3) anterior tip of mandibular symphysis, (4) right caudo-lateral tip of mouth, (5) left caudo-lateral tip of mouth, (6) midpoint on right side of mandible between mandibular symphysis and right caudo-lateral tip, (7) midpoint on left side of mandible between mandibular symphysis and left caudo-lateral tip, (8) hyoid arch, (9) midline joint between left and right branchiostegal rays, (10) caudolateral margin of right operculum, (11) caudolateral margin of left operculum, (12) right pectoral fin base, (13) left pectoral fin base, and (14) anterior tip of pelvic sucker. For lateral view (b), labeled points are as follows: (15) anterior tip of upper lip, (16) anterior edge of upper lip base, (17) caudal tip of junction between maxilla and dentary, (18) anterior edge of neurocranium, (19) center of eye, (20) junction between neurocranium and epaxial muscle insertion, (21) caudal edge of operculum, (22) dorsal edge of pectoral fin base, and (23) ventral edge of pectoral fin base spine.

Kinematic data were extracted from feeding and climbing videos of *S. stimpsoni* by digitizing twenty-three anatomical landmarks across ventral and lateral view footage (Fig. 1), using a modification of the public domain NIH Image program for Macintosh, developed at the US National Institutes of Health (the modification, QuickImage, was developed by J. Walker and is available at http://www.usm.maine.edu/~walker/software). Cycles of each behavior were defined as starting with the first identified forward movement of the premaxilla, and ending with the completion of rearward movement of the premaxilla. Landmarks were digitized for every frame of identified cycles.

**Figure 2:**
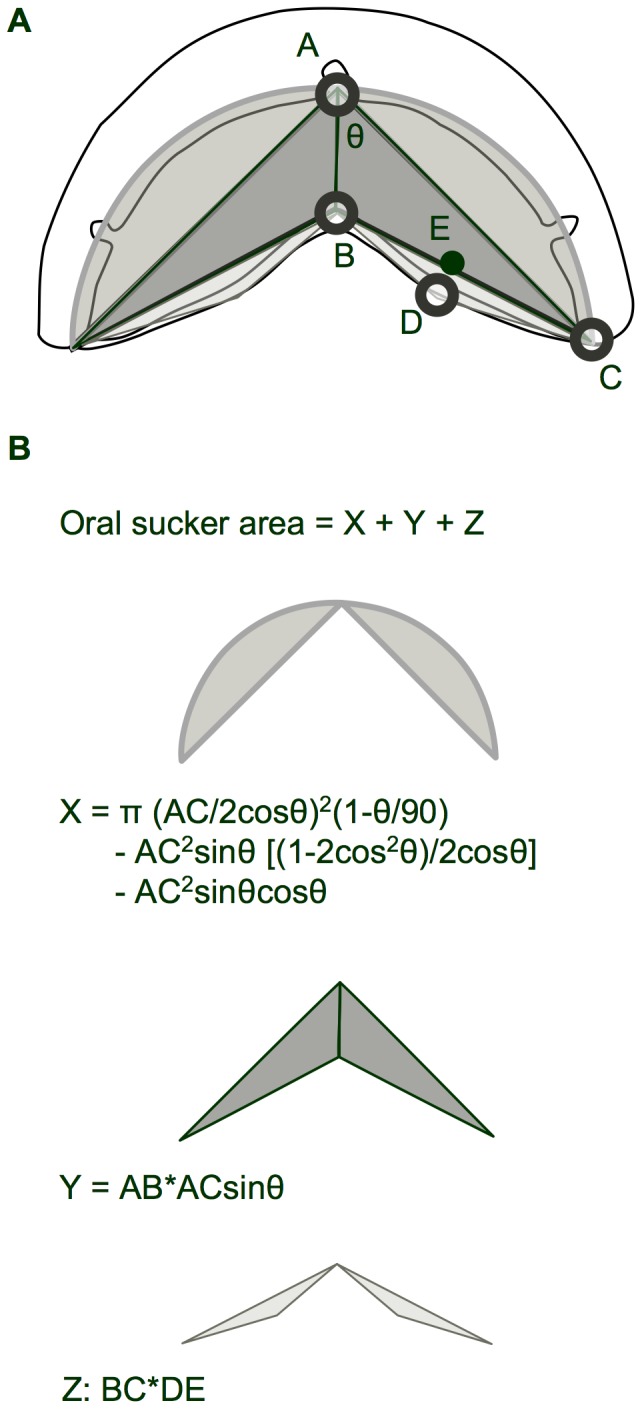
Geometric model for the calculation of oral sucker area from digitized landmarks in ventral view footage of feeding and climbing by *Sicyopterus stimpsoni*. (a) Outline sketch of the mouth of *S. stimpsoni* in ventral view, with superimposed greyscale shaded geometric shapes defined by labeled digitized landmarks. A, digitized Point 1 (anterior edge of upper lip); B, digitized Point 3 (anterior tip of mandibular symphysis); C, digitized Point 4 (right caudolateral tip of mouth); D, digitized Point 6 (midpoint of mandible on the right side); E, calculated midpoint between points B and C; θ, angle between vectors AB and AC. Note that digitized points are shown as open circles, and calculated points are shown as solid circles. (b) Geometric shapes from (a) separated into three groups (designated X, Y, and Z), with formulae for calculation of their areas. Sucker area was modeled as the sum of these three geometric areas, which assume symmetry between left and right sides.

Custom programs written in Matlab (Mathworks, Inc.; Natick, MA) were used to calculate eight kinematic variables from digitized landmark data for each behavior (Table 1, Figs. 1, 2). To facilitate comparisons across individuals of different size, linear measurements were normalized by total body length (BL) and oral sucker area was normalized by BL^2^. QuickSAND software [34] (available at http://www.usm.maine.edu/~walker/software.html) was then used to fit a quintic spline function to the values of each variable for each trial, smoothing the data and allowing all trials to be normalized to the same duration, with values calculated for 101 evenly spaced increments. These smoothed and normalized data were used to calculate average kinematic profiles and standard errors for each variable for both feeding and climbing cycles.

**Table 1:** Description of kinematic variables calculated from landmark data for comparisons of feeding and climbing behaviors by *Sicyopterus stimpsoni.*

Kinematic variable	Description
Cranial elevation angle	Rotation angle of a vector between the anterior edge of the neurocranium (point 18) and the insertion of epaxial muscles on the neurocranium (point 20), relative to the orientation of this vector at the beginning of the cycle
Premaxillary protrusionangle	Rotation angle of a vector between the anterior tip of the upper lip (point 15) and the anterior edge of the neurocranium (point 18), relative to the orientation of this vector at the beginning of the cycle
Premaxillary protrusionlength	Distance that the anterior tip of the upper lip (point 15) has extended during the cycle, relative to its position at the start of the cycle
Hyoid retraction angle	Angle between a vector running from the midpoint of the hyoid arch (point 8) to a stationary point calculated midway between the bases of the left and right pectoral fins (average of points 12 and 13), and a vector running from the midpoint of the hyoid arch (point 8) to the right opercular landmark (point 11)
Hyoid retraction length	Change in the distance between the midpoint of the hyoid arch (point 8) and a stationary point calculated midway between the bases of the left and right pectoral fins (average of points 12 and 13), relative to this distance at the start of the cycle
Mandibular retraction length	Change in distance from the anterior tip of the mandible (point 3) to a stationary point calculated midway between the bases of the left and right pectoral fins (average of points 12 and 13), relative to this distance at the start of the cycle
Opercular expansion length	Distance between left and right opercular landmark tips (points 10 and 11)
Oral sucker area	Geometric model of the area enclosed by the oral sucker in ventral view; see Fig. 2 for calculation

### Statistical Comparisons

Comparisons of feeding kinematics across species, and between feeding and climbing in *S. stimpsoni*, were approached in two ways. First, the peak values of kinematic variables were compared across groups using non-parametric Kruskal-Wallis tests for comparisons across all three study species, and non-parametric Mann-Whitney *U* tests for comparisons between feeding and climbing in *S. stimpsoni* [35]. For comparisons across species, kinematic data could be extracted from Maie et al. [28] for the following variables examined for *S. stimpsoni* in this study: premaxillary protrusion length, hyoid excursion angle, mandibular retraction length (termed “mandibular depression” by Maie et al. [28]), and opercular excursion length, with all lengths normalized to BL. For comparisons between feeding and climbing in *S. stimpsoni*, all eight kinematic variables described in Table 1 were compared. Non-parametric tests were performed using StatView software for Apple Macintosh (Abacus Concepts, Berkeley, CA, USA).

Our second set of comparisons evaluated the similarity of overall kinematic profiles, in addition to maximum values. In these analyses, for each species (or for each behavior in *S. stimpsoni*), the 101 mean values of each variable (calculated for each 1% increment through the kinematic cycle) were used to generate vectors with 101 dimensions. The angle between pairs of these vectors could then be calculated using standard equations [36]–[38]. An angle near 0° indicates two nearly identical vectors (i.e., two nearly identical kinematic profiles), whereas an angle near 90° indicates perpendicular trajectories (profiles that are not correlated, or are independent of each other). These calculations were performed in Microsoft Excel for parallel comparisons to those described for maximum values.

## Results

### Basic Cranial Kinematics of Feeding and Climbing in *Sicyopterus stimpsoni*

**Figure 3:**
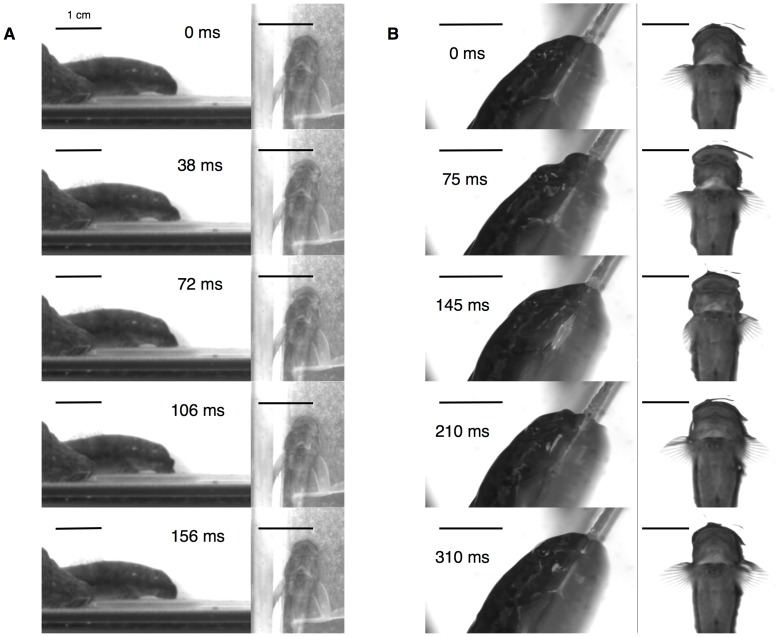
Representative lateral and ventral view still frames from high-speed video of (a) feeding and (b) climbing cycles of *Sicyopterus stimpsoni*. Panels are sequential from top to bottom for each behavior, with elapsed time through the cycle reported in lateral frames. Note in (b) that the fish climbs upwards (toward the top of each frame) as frames are viewed in order from top to bottom. Because climbing cycles are longer in duration than feeding cycles, the five time points illustrated for each behavior represent equivalent fractions of time through the kinematic cycle, at 0%, 25%, 50%, 70%, and 100%. All scale bars equal 1 cm. Note that lateral and ventral views for each behavior are filmed at different magnifications.

**Figure 4:**
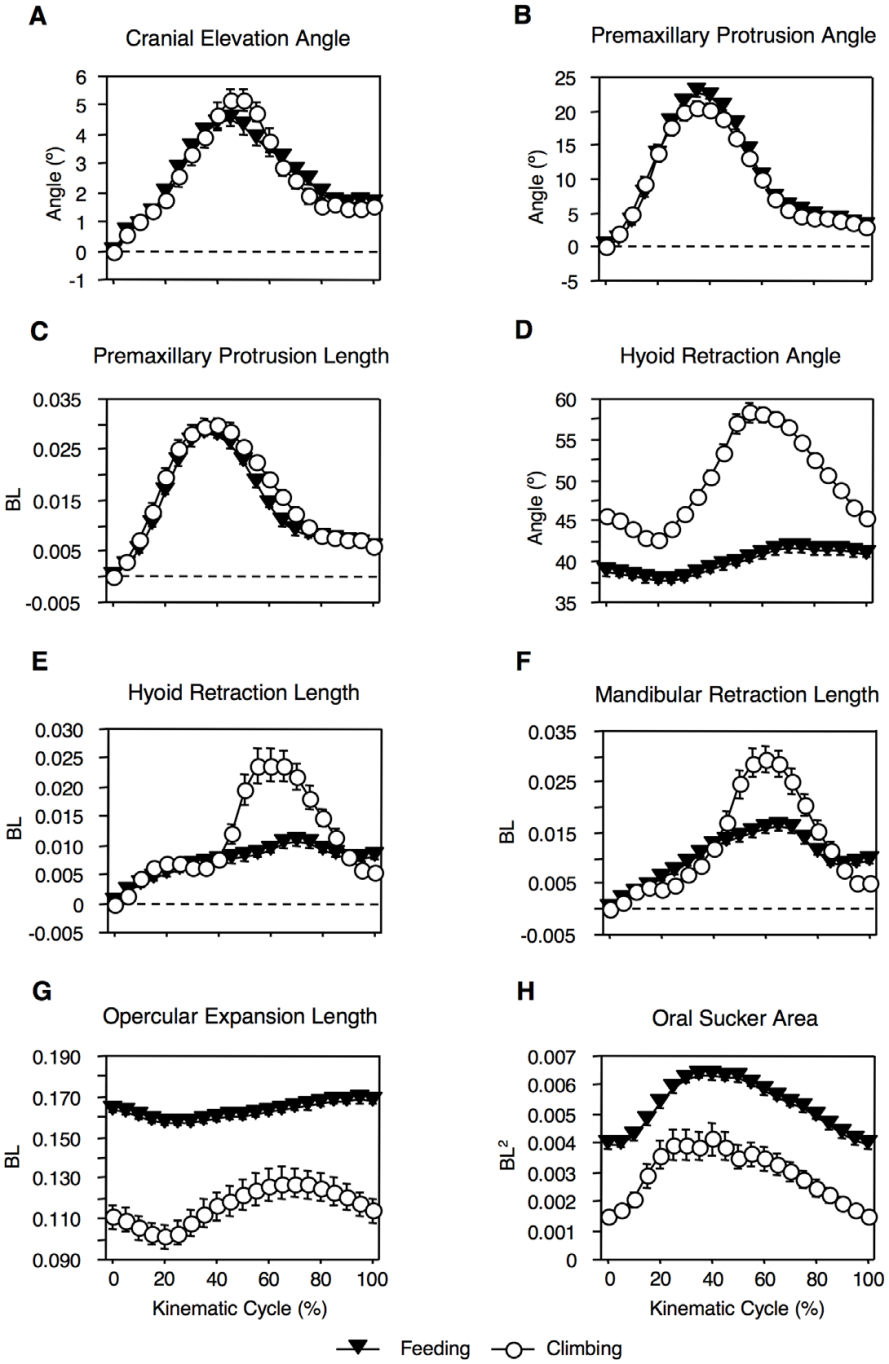
Comparative profiles of cranial kinematics for *Sicyopterus stimpsoni* during feeding (solid triangles) and climbing (open circles) behaviors. Descriptions of the calculation of each variable are provided in the text. Plots show mean (± s.e.m. values of each variable, averaged across all cycles for each behavior for every 5% increment of cycle duration. (a) Cranial elevation angle, (b) premaxillary protrusion angle, (c) premaxillary protrusion length, (d) hyoid retraction angle, (e) hyoid retraction length, (f) mandibular retraction length, (g) opercular expansion length, and (h) oral sucker area. All linear measurements are normalized by body length (BL), or BL^2^ for oral sucker area.

Feeding cycles in *S. stimpsoni* are initiated by forward and dorsal movement of the premaxilla (Fig. 3a). As the premaxilla approaches maximal extension, the mandible and hyoid both retract posteriorly, enlarging the oral sucker area (Figs. 3a and 4). Once maximum gape has been reached, the premaxilla is in contact with the substrate and begins to retract, facilitating the scraping of benthic diatoms from the feeding surface. Surfaces with thick diatom growth typically were visibly cleaner after such episodes.

Climbing cycles in *S. stimpsoni* start similarly to feeding cycles with forward movement of the premaxilla, but in climbing the premaxilla maintains closer contact with the substrate (i.e., with less significant lifting) throughout the cycle (Fig. 3b). Rapid retraction of the mandible and hyoid follow the excursion of the premaxilla, increasing oral sucker area (Fig. 4). Once the mandible, hyoid, and premaxilla motion are complete, the pelvic sucker advances upward to complete the climbing cycle. Climbing cycles last approximately twice as long as feeding cycles (mean ± SE = 0.313±0.009 s for climbing, 0.141±0.002 s for feeding; Mann-Whitney *U*, *P*<0.0001).

### Kinematic Comparisons of Feeding between *S. stimpsoni* and other Hawaiian Gobiids

Comparisons of maximum kinematic values confirm that the benthic scraping behavior of *S. stimpsoni* involves cranial movements that are significantly different from the suction feeding behaviors of *A. guamensis* and *L. concolor* in many respects. Statistical comparisons of mean maxima show that *S. stimpsoni* exhibits the least mandibular retraction and the smallest excursion of the hyoids and opercula among the three species, but the greatest premaxillary protrusion (Table 2). For the variables where *S. stimpsoni* shows the lowest values, its maxima are generally one half or less of the values of other species. In contrast, *S. stimpsoni* exhibits the greatest maximal premaxillary protrusion, but exceeds that of the closest species (*L. concolor*) by only 30% (Table 2).

**Table 2:** Comparison of cranial kinematic data during feeding among the Hawaiian gobiid fishes *Sicyopterus stimpsoni*, *Awaous guamensis*, and *Lentipes concolor* ***,*** showing statistical comparisons of mean maximum values and divergence angles for kinematic profile vectors.

			Divergence angle for kinematic profile vectors (°)	
Kinematic Variable	Species	Mean MaximumValue*	*A. guamensis*–*S. stimpsoni*	*L. concolor*–*S.stimpsoni*
Mandibular	*S. stimpsoni*	0.019±0.001		
Retraction	*A. guamensis*	0.030±0.002	5.77	13.03
Length (BL)	*L. concolor*	0.044±0.002		
Hyoid	*S. stimpsoni*	4.710±0.357		
Retraction	*A. guamensis*	8.206±0.822	3.74	2.98
Angle (°)	*L. concolor*	14.036±0.659		
Opercular	*S. stimpsoni*	0.007±0.001		
Expansion	*A. guamensis*	0.035±0.002	4.24	3.03
Length (BL)	*L. concolor*	0.044±0.001		
Premaxillary	*S. stimpsoni*	0.031±4.570×10^−4^		
Protrusion	*A. guamensis*	0.016±0.001	45.31	46.93
Length (BL)	*L. concolor*	0.024±0.001		

Data for *A. guamensis* and *L. concolor* derived from [28].

For *S. stimpsoni*, *N* = 95 cycles, 4 individuals; for *A. guamensis*, *N* = 28 cycles, 3 individuals; for *L. concolor*, *N* = 33 cycles, 3 individuals.

* All interspecific comparisons significant at *P*<0.0001 (non-parametric Kruskal-Wallis tests).

In contrast to comparisons of maximum kinematic values, overall patterns of motion throughout the cycle for several variables are fairly similar across the species. In particular, for the three variables for which *S. stimpsoni* showed the lowest maxima, five of the six interspecific comparisons of overall kinematic profiles showed divergence angles of less than 6°, and the sixth (mandibular retraction length between *L. concolor* and *S. stimpsoni*) showed a divergence of only 13° (Table 2). However, for premaxillary protrusion, overall profiles of motion show more dramatic differences than for other variables, with divergence angles between *S. stimpsoni* and both suction feeding gobiids exceeding 45° (Table 2). This difference appears to be driven by the more cyclic motion of the premaxilla in *S. stimpsoni*. In *S. stimpsoni*, the premaxilla returns nearly to its starting position at the end of a feeding cycle as the teeth scrape rearward along the substrate (Figs. 3a and 4c), whereas the premaxilla often remains in a fairly extended position after the jaws close during suction feeding in *A. guamensis* and *L. concolor* ([28]: Figs. 4, 5c).

### Kinematic Comparison of Feeding and Climbing in *S. stimpsoni*

Similar to comparisons across species, maximum values of kinematic variables between feeding and climbing are significantly different for each of the variables we compared, although many differences are fairly small in magnitude (Table 3). The greatest differences (ranging from 29–100%) are between hyoid retraction angle and length and mandibular retraction length (for which climbing shows larger values), and for opercular expansion length (for which feeding shows larger values). Peak oral sucker area also was moderately greater in feeding than in climbing. In contrast, maxima for premaxillary variables (though significantly different) differed by only 9–13% between behaviors.

**Table 3:** Comparison of cranial kinematic data between climbing and feeding for *Sicyopterus stimpsoni* ***,*** showing results of Mann-Whitney *U* tests for mean maximum values, and divergence angles for kinematic profile vectors.

	Maximum values			
Kinematic Variables	Feeding(*N* = 96 cycles)	Climbing(*N* = 36 cycles)	P-Values*	Divergence angle for kinematic profile vectors (°)
Cranial elevation angle (°)	5.824±0.234	7.173±1.858	0.0004	7.56
Premaxillary protrusion angle (°)	25.031±0.384	22.897±0.705	0.0319	3.60
Premaxillary protrusion length (BL)	0.031±4.570×10^−4^	0.035±0.001	0.0064	4.23
Hyoid retraction angle (°)	43.408±0.525	61.131±1.016	<0.0001	4.85
Hyoid retraction length (BL)	0.014±0.001	0.028±0.003	<0.0001	20.96
Mandibular retraction length (BL)	0.019±0.001	0.034±0.002	<0.0001	17.70
Opercular expansion length (BL)	0.171±0.001	0.132±0.008	0.0128	3.65
Oral Sucker Area (BL^2^)	0.007±9.617×10^−5^	0.005±0.001	0.0382	9.58

* All Mann-Whitney *U* tests indicate significant differences between feeding and climbing at P<0.05.

In another parallel with comparisons of feeding across species, significant differences in mean maximum values of variables between feeding and climbing did not necessarily correspond to large divergence angles for their kinematic profiles. Four variables showed profiles differing by less than 5° and six by less than 10° (Table 3), with an average (± s.e.m.) of 9.0±2.4° across all variables. The greatest divergences were for the profiles of hyoid retraction length (20.96°) and mandibular retraction length (17.70°). In both of these variables, climbing cycles showed a steeper increase to a greater peak value at midcycle, followed by a closer return to starting position at the end of the cycle (Fig. 4e–f).

## Discussion

### Distinctiveness of Benthic Scraping Kinematics in *S. stimpsoni* Compared to Suction Feeding in other Gobiids

Given that *S. stimpsoni* consumes a different diet and employs a different feeding behavior than other Hawaiian gobiids (benthic scraping versus suction feeding), it is not surprising that feeding kinematics should differ across these species. However, our kinematic comparisons establish the specific sets of movements that differ between these behaviors, clarifying what changes in function facilitated the novel acquisition of scraping as a mode of feeding. In general, benthic scraping in *S. stimpsoni* involves extreme protrusion of the premaxilla with limited retraction of the hyoid and mandible; in contrast, both forward protrusion of the premaxilla and substantial rearward retraction of the mandible and hyoid contribute to rapid jaw opening during suction feeding in other gobies (Table 2; [28]). The substantially greater expansion of the opercula in suction feeding species (Table 2) matches functional expectations, as it could contribute to the expansion of intracranial volume required to generate negative pressures that draw in water (and food) during suction feeding (e.g., [39]). The retention of slight opercular expansion (as well as moderate mandibular and hyoid retraction) during feeding in *S. stimpsoni* might reflect retention of ancestral traits, and potentially allow a degree of suction that could help draw food into the mouth once it has been scraped off the substrate.

Despite exhibiting significant differences in the maximum values of several kinematic variables, in most cases *S. stimpsoni* showed kinematic profiles that were very similar (Table 2) to those from both suction feeding gobiids from which data were available for comparison [28]. This indicates that, for many types of motion, differences between benthic scraping and suction feeding could be viewed as changes in the extent of motion, rather than reflections of fundamentally different kinematic patterns. Premaxillary movements were the exception to this pattern (Table 2) and, among the variables we examined, showed the greatest difference that might characterize the distinction between scraping and suction kinematics. In fact, the differences in premaxillary profiles between scraping and suction feeding goby species were greater than the differences in premaxillary profiles between feeding and climbing in *S. stimpsoni*, which were some of the most similar among the variables compared between the two behaviors (Tables 2 and 3). This raises an interesting possibility that premaxillary movements might somehow be constrained in *S. stimpsoni* (and potentially other *Sicyopterus* species) once they diverged from the ancestral pattern, limiting their potential to vary across behaviors. Alternatively, it is possible that additional premaxillary protrusion could be functionally disadvantageous for feeding, climbing, or both. During feeding, increased gape resulting from additional premaxillary protrusion might weaken suction that would help retain scraped diatoms in the oral cavity, rather than being swept away by flow. During climbing, further increases in gape might impede the generation of negative pressure required for use of the mouth during adhesion [11]. Further anatomical comparisons of the premaxillary apparatus across gobiids could help to assess the role of structural or functional constraints in the patterns observed.

### Oral Mechanics of Feeding Versus Climbing in *S. stimpsoni*

Some differences in cranial kinematics between feeding and climbing in *S. stimpsoni* can be interpreted in light of the different functional requirements of these behaviors. For example, opercular expansion is smaller throughout the cycle during climbing compared to feeding (Fig. 4g). This should help to make the head of the fish narrower in the face of oncoming water, potentially increasing streamlining and reducing drag that the fish would have to resist to avoid dislodgement [31], [32].

Other differences observed between feeding and climbing kinematics in *S. stimpsoni* were unexpected. It is possible that some of these differences may reflect consequences of the direction of animal motion during the behavior, rather than any functional advantage. For example, one factor contributing to the greater peak retraction magnitudes of the mandible and hyoid during climbing may be the advancement of the anterior portion of the head up the climbing slope [9]. Linkage mechanics of cranial elements could force retraction of the mandibular and hyoid elements as the whole head narrows during the portion of climbing during which the head advances [40].

Another unexpected difference between feeding and climbing kinematics might reflect a passive consequence of the difference in body orientation between these behaviors, rather than any functional advantage during either behavior. Because adhesive capacity relates to the size of the sucker [11], greater oral sucker area might have been expected during climbing; instead, it was greater during feeding throughout the cycle (Fig. 4h). During feeding the fish is horizontal and can press its mouth down on the substrate during scraping, spreading the area of the sucker. In contrast, during climbing the body is largely out of water, experiences gravitational pull, and is climbing up a steeply angled surface (Fig. 3). As body weight pulls the fish downwards it produces a turning moment about the lowest point of body contact with the substrate, similar to a picture hung on a wall [41]. Once the angled surface is sufficiently steep, this could pull the fish away from the substrate, pulling up on the sucker and reducing its area of contact. Future measurements of feeding on an inclined surface could help to evaluate this possibility, though such behavior could not be successfully elicited during this study.

### Assessing Exaptation in the Cranial Kinematics of *Sicyopterus*

Although *S. stimpsoni* showed statistically significant differences between feeding and climbing for maximum values of each of the eight kinematic variables we evaluated (Table 3), in many cases these differences were small in magnitude, and overall profiles of motion throughout the cycle matched very closely (Table 3, Fig. 4). For three variables in particular (cranial elevation angle, premaxillary protrusion angle, and premaxillary protrusion length) both the values and patterns of motion were so similar that kinematic profile plots for the two behaviors are nearly completely superimposed (Fig. 4a–c). This combination of both similarities and differences between feeding and climbing kinematics complicates the assessment of whether one of these behaviors might represent an exaptation of the other.

However, strict similarity between feeding and climbing kinematics might not be a fair expectation, even if exaptation had occurred. Given the strength of selection that appears to operate on both feeding and climbing performance in climbing gobiids [31], [32], it may not be reasonable to expect patterns for one behavior to remain completely unchanged after being applied to another function. From this perspective, the similarities we observed between feeding and climbing kinematics in *S. stimpsoni* might instead reflect evidence of “exaptation with modifications.” Some key features that are distinct from those of related species (e.g., premaxillary movements) would remain similar between the behaviors. Other features that diverge between behaviors might show different maxima, but without fundamentally different patterns of motion (e.g., opercular expansion) – and, in some cases, the differences observed might be a passive consequence of the conditions under which the behavior is executed, rather than adaptation related to a new function (e.g., oral sucker area).

Beyond assessing the potential for exaptation to have occurred in *S. stimpsoni*, there is little basis for evaluating whether oral kinematics for climbing may have been coopted from feeding, or feeding kinematics coopted from climbing. Given that all gobies use the mouth to feed, but not all use the mouth to climb, the adoption of feeding kinematics toward climbing might be viewed as more likely; however, data on specific character state transformations that would support this conclusion are not available. A conclusion that exaptation may have operated in this system should, itself, be viewed as preliminary. Nonetheless, data from *S. stimpsoni* provide a foundation for additional comparisons of feeding and climbing kinematics in other species of *Sicyopterus* that, when placed in the context of the phylogeny of the genus [16], could indicate the sequence of transformations in oral function that occurred as this lineage diverged and adopted its novel behaviors.
